# Characterization of the Bronchoalveolar Lavage Fluid by Single Cell Gene Expression Analysis in Healthy Dogs: A Promising Technique

**DOI:** 10.3389/fimmu.2020.01707

**Published:** 2020-07-30

**Authors:** Aline Fastrès, Dimitri Pirottin, Laurence Fievez, Thomas Marichal, Christophe J. Desmet, Fabrice Bureau, Cécile Clercx

**Affiliations:** ^1^Department of Clinical Sciences, Faculty of Veterinary Medicine, FARAH, University of Liège, Liège, Belgium; ^2^Laboratory of Cellular and Molecular Immunology, Department of Functional Sciences and GIGA-Inflammation, Infection and Immunity, University of Liège, Liège, Belgium

**Keywords:** single-cell RNA-sequencing, dog, bronchoalveolar lavage fluid, cell, lung

## Abstract

Single-cell mRNA-sequencing (scRNA-seq) is a technique which enables unbiased, high throughput and high-resolution transcriptomic analysis of the heterogeneity of cells within a population. This recent technique has been described in humans, mice and other species in various conditions to cluster cells in populations and identify new subpopulations, as well as to study the gene expression of cells in various tissues, conditions and origins. In dogs, a species for which markers of cell populations are often limiting, scRNA-seq presents with elevated yet untested potential for the study of tissue composition. As a proof of principle, we used scRNA-seq to identify cellular populations of the bronchoalveolar lavage fluid (BALF) in healthy dogs (*n* = 4). A total of 5,710 cells were obtained and analyzed by scRNA-seq. Fourteen distinct clusters of cells were identified, further identified as macrophages/monocytes (4 clusters), T cells (2 clusters) and B cells (1 cluster), neutrophils (1 cluster), mast cells (1 cluster), mature or immature dendritic cells (1 cluster each), ciliated or non-ciliated epithelial cells (1 cluster each) and cycling cells (1 cluster). We used for the first time in dogs the scRNA-seq to investigate cellular subpopulations of the BALF of dog. This study hence expands our knowledge on dog lung immune cell populations, paves the way for the investigation at single-cell level of lower respiratory diseases in dogs, and establishes that scRNA-seq is a powerful tool for the study of dog tissue composition.

## Introduction

The cells can be considered as the fundamental unit in biology. They are working in concert to respond to stimuli in order to maintain health. However, until recently, they were only characterized and distinguished using microscopy-based methods, flow cytometry, or bulk RNA sequencing, all techniques that are quite limiting for demonstration of cell heterogeneity (1–4). With the development of next-generation sequencing technologies, it is now possible to profile the transcriptome of each individual cell composing a sample. The single-cell mRNA sequencing (scRNA-seq) enables high throughput and high-resolution transcriptomic analysis of the cellular heterogeneity with an unbiased assessment of the cells as it gives the opportunity to identify cells without relying on previously known cell markers. It has become a powerful tool to identify cell subpopulations sharing similar transcriptome within a population, as well as to provide information related to cell fate, development, lineage, physiology, homeostasis and underlying molecular mechanisms (2, 5, 6). The use of this recent technique has been described in humans (7–9), mice (10) and other species (11, 12) in various conditions and samples. In dogs, the use of this technique has not yet been reported so far.

Bronchoscopy and combined analysis of the bronchoalveolar lavage fluid (BALF) are largely used in the diagnosis of canine lower airway diseases either acute or chronic (13, 14). In dogs, bronchoalveolar lavage is a well-tolerated procedure and few adverse effects are reported (13, 14). Common analyses performed on BALF include determination of total (TCC) and differential cell counts (DCC) (including macrophages, neutrophils, lymphocytes, eosinophils, and mast cells count), cytological examination of cytospin preparations, bacterial cultures and detection of specific respiratory pathogens using quantitative polymerase chain reactions (13, 14). Only few studies have characterized the lymphocyte populations in the canine BALF by flow cytometry (15–19) while the other cell types have not been studied. In depth examination of BALF cellular composition and subpopulations as well as the comparison of these cell subpopulations in healthy and diseased conditions could lead to the identification of new cell subsets involved in disease and could help to better understand the pathophysiology of lung diseases, the cell adaptations in disease context as well as to find new or more specific therapeutic targets (6, 20).

In this study, we aimed to use the scRNA-seq technique in healthy client-owned dogs to analyze BALF cell subpopulations. Results will contribute to provide a base resource regarding cell subpopulations composing the BALF of healthy dogs which could be of great interest for further investigations of the BALF cell subpopulations in disease.

## Materials and Methods

### Dog Population

For the scRNA-seq analysis, BALFs were obtained from healthy dogs prospectively recruited at the veterinary clinic of the University of Liège (CVU, Liège, Belgium) between December 2017 and June 2018. All dogs were privately owned, and samples were obtained with owners' consent. The study was validated by the ethical committee of the University of Liège (approval no. 1435).

The healthy status of the dogs was confirmed by history, normal physical examination, blood work (plasma biochemistry and hematology), bronchoscopy and analysis of the BALF (including a macroscopic evaluation, a TCC and a DCC). Dogs from various breed and age were chosen to better represent the diversity of the canine population.

### Samples Collection

BALFs were obtained under anesthesia with butorphanol at 0.2 mg/kg (Butomidor®, Richter Pharma AG, Wels, Austria) as premedication and propofol (Diprivan®, Asen Pharma Trading Limited, Dublin, Irland) infusion on demand.

Dogs were not intubated for the procedure. A bronchoscope (FUJINON© Pediatric Video-Bronchoscope EB-530S), cleaned and disinfected using the washer-disinfector Serie 4 (Soluscope®, Aubagne, France), was inserted into the bronchi until the extremity was wedged. Three to four mL/kg of a sterile NaCl 0.9% solution divided into 3 aliquots were instilled through the endoscope channel into the lung (2 aliquots were obtained in the right diaphragmatic lobe and one in the left diaphragmatic lobe) and directly reabsorbed by gentle suction into the same sterile recipient. About 1 mL of BALF was kept for total and differential cell count calculation performed using, respectively, a hemocytometer and a cytospin preparation (centrifugation at 221 g, for 4 min at 20°C, Thermo Shandon Cytospin©4), by counting a total of 200 cells at high power field. The rest of the BALF was then transferred within 15 to 20 min following collection on ice to the GIGA laboratory of Cellular and Molecular Immunology.

### Single-Cell RNA Sequencing

#### BALF Samples Preparation

BALFs were filtered to remove mucus and total cell count was assessed using a hemocytometer and Türk coloration (Supplementary Table 1). BALFs were then centrifuged at 400 g for 7 min and the pellet resuspended in phosphate-buffered saline solution (Gibco^TM^ 1x DPBS, Cat.14190-169) to obtain a cell concentration around 1,000 cells/μL. A second filtration through a cell strainer (BD Falcon™, Biosciences, USA, Cat.352350) was performed to remove any remaining cell debris and large clumps and cells were again counted with Trypan blue staining to assess cell viability considered as acceptable above 70% (Supplementary Table 1). The volume of the cell suspension was then adjusted to obtain a final cell concentration between 500 and 1,000 cells/μL suspended in phosphate-buffered saline solution containing 0.04% (w/v) bovine serum albumin (Supplementary Table 1).

For each sample, ~3,500 cells (Supplementary Table 1) were loaded into the Chromium^TM^ Controller (10x Genomics, Pleasanton, CA, USA) ~30 min after the first filtration and were then partitioned into nanoliter scale vesicles containing 10x barcoded beads from Chromium^TM^ Single Cell 3′ Gel Bead Kit v2 (10x Genomics, Pleasanton, CA, USA) according to manufacturer's instructions. The following steps take place in the vesicles containing cell: [1] cell lysis, [2] capture of polyadenylated mRNAs oligonucleotides containing cell specific 16 bp barcode and 10 bp Unique Molecular Identifier (UMI) and [3] reverse transcription of mRNAs into cell specific barcoded cDNAs on a Veriti© 96-Well Thermal Cycler (ThermoFisher Scientific, Merelbeke, Belgium).

#### Single-Cell Library Preparation and Sequencing

Emulsion breakage, cDNA amplification and libraries construction were performed using Chromium^TM^ Single Cell 3′ Reagent kit v2 (10x Genomics, Pleasanton, CA, USA) according to manufacturer's instructions as already described (21). Briefly, cDNAs obtained were amplified in a Veriti© 96-Well Thermal Cycler. Amplified cDNA products were cleaned up, quality controlled and quantified. Illumina's P5, P7, and Read2 primers, as well as Sample Index were then added to generate sequencing libraries. The barcoded sequencing libraries were also quality controlled and quantified by quantitative PCR (KAPA Biosystems Library Quantification Kit for Illumina platforms). Sequencing libraries were loaded on an Illumina NextSeq500. The sequencing depth was set at 50,000 reads per cell, taking into account that ~2,000 cells should be captured (55–60% efficiency). Cell Ranger software (v1.2.0) (10x Genomics, Pleasanton, CA, USA) was used to demultiplex Illumina BCL files to FASTQ files (cellranger mkfastq), to perform alignment to dog genome (CanFam3.1, GenBank assembly accession: GCA_000002285.2), filtering, UMIs counting and to produce gene-barcode matrices (cellranger count).

#### Data Analysis and Visualization

Analyses were performed using R package Seurat (version 3.1.2) (22). Briefly, we have first selected cells with a minimum of 100 and a maximum of 2,500 unique mapped genes to exclude low-quality cells or empty droplets and cell doublets or multiplets, respectively. Only genes present in at least 3 different cells were kept. Expression values were normalized to 10,000 transcripts per cell and the “FindVariableFeatures” function was used to identify the top 2,000 variable genes in each BALF sample. “FindIntegrationAnchors” and “IntegrateData” functions were used to combine the data of all BALF specimens, while minimizing batch effects. Next, a linear transformation using the “ScaleData” function was applied so that highly-expressed genes do not dominate. A principal component analysis (PCA) was performed on the scaled data using the command “RunPCA.” The statistically principal components taken into account for the next analysis were identified using the “PCElbowPlot” and the “DimHeatmap” functions and were set to 1:30. A K-nearest neighbor graph, based on the Euclidean distance in PCA space and the Jaccard similarity index, was obtained using the “FindNeighbors” function. Cells were then clustered with the “FindClusters” command based on the Louvain algorithm. Several cluster resolutions were tested, and the resolution of 0.3 was chosen, since higher resolutions created additional subdivisions of non-well-defined clusters or clusters containing singlets, which were considered not biologically relevant. The data were visualized by a non-linear dimensional reduction, the t-distributed stochastic neighbor embedding (t-SNE) plots, using the “RunTSNE” function, with the number of dimensions to use set to 30 (PC 1:30).

Cell types within each cluster were characterized based on the identification of differentially expressed genes (DEGs) specific for each cluster compared to all others. The “FindMarkers” function was used to identify DEGs across clusters. Clusters with the same identified cell type were also further characterized by comparing DEGs between each other. The differential expression was measured using non-parametric Wilcoxon rank sum tests adjusted for multiple testing with Bonferroni correction. Only DEGs with an adjusted *P* < 0.05 were retained. The genes not well-annotated were further blasted on the Ensembl genome browser (v99.31) (23) for dog species to increase the annotation rate. Specific cell markers average expression and percentage of cells expressing the indicated genes within clusters were visualized with the “DotPlot” function. Alternatively, the “FeaturePlot” function was used to show specific gene expression within single cells.

The different common biological processes between clusters with the same identified cell type were also assessed using the gene set enrichment analysis (GSEA) using the online GSEA-P software (24). GSEA was carried out by computing overlaps between significantly enriched genes calculated between clusters with the same identified cell type and gene ontology (GO) biological process gene sets using hypergeometric tests with Benjamini Hochberg correction for multiple testing (*P*-value adjusted). Only the 10 first gene sets that best overlapped with our gene set were retained.

### Statistical Analysis

Single-cell mRNA sequencing data from the 4 samples were pooled for all analysis. A *P*-value lower than 0.05 was considered as significant. Details about statistical analysis for the scRNA-seq data and the gene set enrichment analysis can be found in the “Data analysis and visualization” section above.

## Results

### Dogs Population Characteristics

Four healthy client-owned dogs were recruited for the bronchoalveolar lavage procedure. The cohort was exclusively composed by adult females including one 4-year-old French bulldog, one 6-year-old Australian shepherd, one 9-year-old West Highland white terrier and one 11-year-old Yorkshire terrier.

### BALF Cells Analysis

Information about TCC and DCC for each BALF can be found in the Table 1.

**Table 1:** Total and differential cell count in each bronchoalveolar lavage fluid.

		**BALF 1**	**BALF 2**	**BALF 3**	**BALF 4**
TCC, cells/μL		440	880	570	180
DCC, %	Macrophages	70	80	91	71
	Neutrophils	10	12	3	12
	Lymphocytes	18	5	3	10
	Eosinophils	1	3	1	2
	Mast cells	0	0	0	0
	Epithelial cells	1	0	2	5

*BALF 1, female Yorkshire terrier of 11-year-old; BALF 2, female French bulldog of 4-year-old; BALF 3, female West Highland white terrier of 9-year-old; BALF 4, female Australian shepherd of 6-year-old; TCC, total cell count; DCC, differential cell count*.

### Single-Cell RNA Sequencing

The transcriptomic profile from a total of 5,710 cells was obtained from each of the four BALF specimens using 10x Genomics based scRNA-seq analysis. Cells had a mean read depth of ~54,000 reads per cell. Summary of sequencing and mapping quality control metrics for each BALF sample is presented in Table 2. The distribution of transcripts and genes counts can be found in the Supplementary Figures 1A,B.

**Table 2:** Metrics about mapping and characteristics of the detected cells of each BALF sample.

	**BALF 1**	**BALF 2**	**BALF 3**	**BALF 4**
Number of cells passing quality control	1,309	1,072	1,298	2,031
Reads mapped confidently to genome, %	68.1	68.6	59.8	72.4
Reads mapped confidently to transcriptome, %	26.5	30.4	23.9	30.7
Median genes/cell	485 (229–2,480)	780 (350–1,313)	834 (376–1,046)	407 (215.25–953)
Median UMIs/cell	1020 (321–3,351)	1942 (720–4,003)	1888.5 (678–2,671)	837 (430–2,669)
Total genes detected	11,343	11,133	10.839	11,543

*Data were generated after passing quality control including the exclusion of cells with <100 and >2,500 genes. Reads mapped confidently to genome are the number of reads that mapped only to the genome. Reads mapped confidently to transcriptome are the fraction of the reads mapped to a unique gene in the transcriptome and are considered for UMI counting. Median genes per cell correspond to the median number of genes with at least one UMI count. Total genes detected is the detected number of genes with at least one UMI count in any cell. BALF 1, female Yorkshire terrier of 11-year-old; BALF 2, female French bulldog of 4-year-old; BALF 3, female West Highland white terrier of 9-year-old; BALF 4, female Australian shepherd of 6-year-old; UMI, unique molecular identifier*.

**Figure 1:**
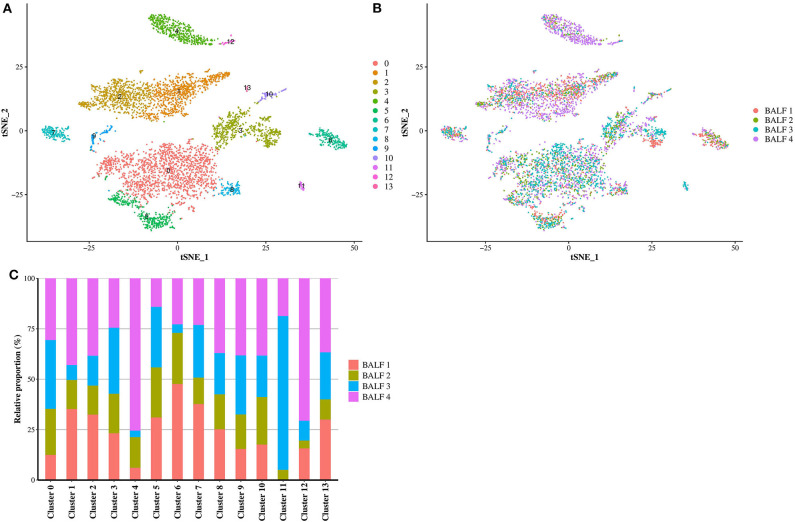
Compiled t-SNE plot of the cell clusters. **(A)** t-SNE plot of all cells (*n* = 5,710) representing the cell clusters analyzed by scRNA-seq. Each color corresponds to one cluster assigned via the graph-based clustering method with a resolution of 0.3. **(B)** Batch alignment across bronchoalveolar lavage fluid (BALF) specimens, each color representing the cells coming from one sample. **(C)** Bar plot showing the relative proportion of the cell from each BALF sample into each cluster. BALF 1, female Yorkshire terrier of 11-year-old; BALF 2, female French bulldog of 4-year-old; BALF 3, female West Highland white terrier of 9-year-old; BALF 4, female Australian shepherd of 6-year-old.

Cells from all dogs were compiled after identification of anchors using Seurat. The clustering in Seurat allowed the detection of 14 well-defined clusters (Figure 1A). The contribution of each individual sample in the compiled t-SNE figure is displayed in Figures 1B,C. Cells coming from each of the four BALF specimens were present in all identified clusters except for the cluster 11 which did not contain cells from BALF 1 (Figure 1C). The average expression of all transcripts detected by clusters is provide in the Supplementary Table 2.

**Figure 2:**
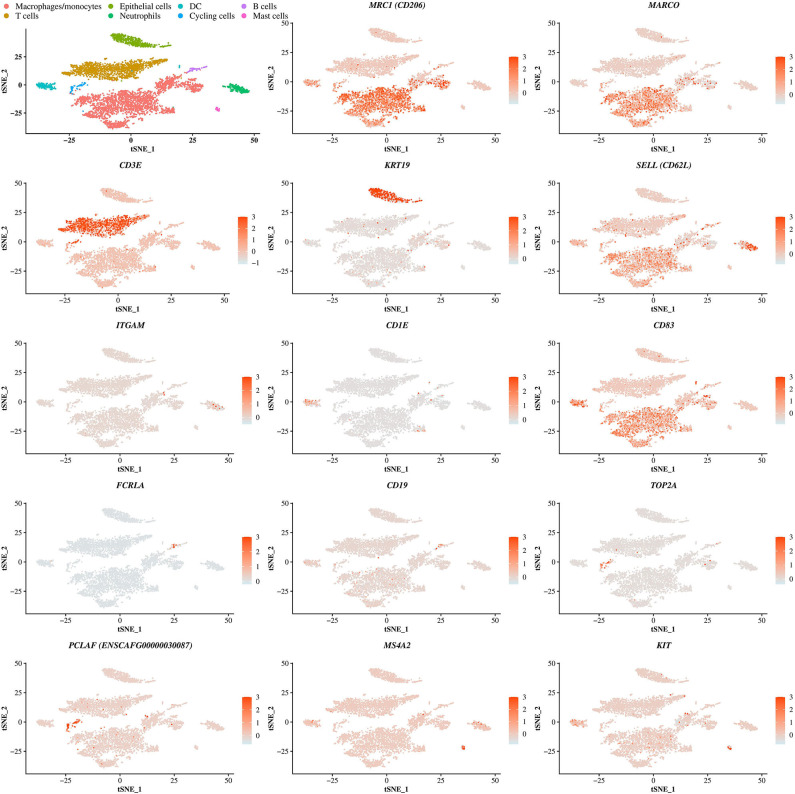
Identification of cell identity corresponding to the clusters. t-SNE plot showing the cells identity based on the expression of differentially expressed genes representative of each cells type including genes coding for the macrophage receptor with collagenous structure (*MARCO*), the macrophage mannose receptor (*MRC1*, encoding *CD206*), the T-cell surface glycoprotein CD3 epsilon chain (*CD3E*), the cytokeratin 19 (*KRT19*), the selectin (*SELL*, encoding *CD62L*), the integrin alpha M (*ITGAM*), the T-cell surface glycoprotein CD1e (*CD1E*), the *CD83* molecule, the Fc receptor like A (*FCRLA*), the *CD19* molecule, the DNA topoisomerase II alpha (*TOP2A*), the proliferating cell nuclear antigen clamp associated factor (ENSCAFG00000030087, encoding *PCLAF*), the membrane spanning 4-domains A2 (*MS4A2*) and the mast/stem cell growth factor receptor (*KIT*). DC, dendritic cell.

The cell identity of each cluster was determined based on the DEGs in each cluster compared to all others. All DEGs are reported in the Supplementary Table 3. In each cluster, a selection of the most overexpressed transcripts able to differentiate cell types according to the literature is displayed in Table 3. Cells of clusters 0, 3, 5, and 8 expressing *MARCO* and/or *MSR1* and/or *HLA-DRB1* and/or *CD163* and/or *CD86* and/or *MRC1* and/or *CD68* and/or *CD63* were identified as macrophages/monocytes (25–32). Cells of cluster 1 and 2 expressing *CD3* markers were identified as T lymphocytes (28, 33). Cells of clusters 4 and 12 expressing *TFF1, TFF3* and *KRT19* or just *KRT19*, respectively, were identified as epithelial cells (34, 35). Cells of clusters 7 and 13 expressing *CD83* and either *CD1E* or CCR7, respectively, were identified as dendritic cells (DC). Finally, cells of cluster 6 expressing *CD62L* and *ITGAM* were identified as neutrophils (36), cells of cluster 9 expressing *PCLAF, TOP2A* and *Ki-67* as cycling cells (8), cells of cluster 10 expressing *BCR, FCRLA* and *CD19* as B lymphocytes (37–39) and finally, cells of cluster 11 expressing *MS4A2, FCER1G, KIT* and *CD63* as mast cells (40) (Table 3 and Figure 2). The proportions of the different identified cell types in the global dataset corresponded to 50.4% of macrophages/monocytes, 28.9% of lymphocytes B and T, 9.5% of epithelial cells, 4.1% of neutrophils, 3.9% of DC 2.2% of cycling cells and 1.0% of mast cells. Of note, we were not able to identify eosinophils, cells known to be present in BALF (13).

**Table 3:** Selection of significant DEGs able to differentiate cell type in each cluster based on literature.

		**Cluster 0**		**Cluster 1**		**Cluster 2**		**Cluster 3**		**Cluster 4**		**Cluster 5**		**Cluster 6**		**Cluster 7**		**Cluster 8**		**Cluster 9**		**Cluster 10**		**Cluster 11**		**Cluster 12**		**Cluster 13**	
		**Avg logFC**	**pct**	**Avg logFC**	**pct**	**Avg logFC**	**pct**	**Avg logFC**	**pct**	**Avg logFC**	**pct**	**Avg logFC**	**pct**	**Avg logFC**	**pct**	**Avg logFC**	**pct**	**Avg logFC**	**pct**	**Avg logFC**	**pct**	**Avg logFC**	**pct**	**Avg logFC**	**pct**	**Avg logFC**	**pct**	**Avg logFC**	**pct**
Macro-phages/Mono-cytes markers	*MARCO*	**1.03**	83	−1	48	−0.99	60	**0.28**	25	−0.97	0	**0.35**	94	−0.97	51	−0.95	38					−0.91	20	−0.96	0	−0.94	24	−0.96	27
	*MSR1*	**0.99**	88	−1.23	46	−1.2	59	**0.63**	35	−1.2	16					−1.24	17									−1.22	20	−1.22	27
	*HLA–DRB1*	**0.85**	100	−1.18	84	−1.5	87			−3.27	26			−2.47	76	**1.33**	100	**0.63**	100					−3.25	19	−2.23	22	−2.1	73
	*CD163*	**0.7**	87	−1.31	24	−1.28	20	**1.17**	45	−1.25	14			−1.13	39							−1.22	16						
	*CD86*	**0.66**	86	−1.27	52	−1.16	59	**0.86**	57	−1.31	0			−1.03	52	**0.76**	91	**0.4**	79	−0.80	50			−1.25	76	−1.22	16		
	*MRC1*	**0.6**	91	−1.68	44	−1.69	54			−1.71	12	−0.52	92	−1.68	65			**0.43**	83			−1.51	43			−1.67	26	−1.6	20
	*CD68*	**0.97**	86	−1.19	36	−1.16	54			−1.18	0	**0.84**	96									−1.13	27	−0.79	3	−1.01	18		
	*CD63*	**0.85**	99	−0.98	64	−1.17	68	−0.26	49	−1	30	**0.39**	100	−0.5	43			**0.32**	97			−1.12	72	**1.76**	97	−0.51	33		
DC markers	*CD1E*															**0.87**	79	**0.26**	55										
	*CD83*	**0.26**	71	−1.06	7	−1.08	16			−0.78	17					**0.57**	83					−0.57	27			−1.04	8	**2.63**	100
	*CCR7*																											**3.24**	93
T cells markers	*CD3E*	−1.75	58	**1.37**	77	**1.54**	87			−1.56	4			−1.57	55	−1.43	25			**0.26**	73								
	*CD3D*	−1.6	56	**1.41**	72	**1.39**	83			−1.32	2	−1.39	55			−1.27	39									−1.39	22		
Epithe-lial cells markers	*TFF3*			−3.92	61	−4.15	67	−3.29	79	**6.18**	99	−4.17	93	−3.5	54							−3.24	77					−4.46	67
	*TFF1*			−3.31	58	−3.42	64	−2.31	78	**5.4**	95	−3.3	93	−2.66	77									−3.82	81	−3.39	28		
	*KRT19*	−1.84	60					−1.15	67	**3.32**	81			−1.66	52	−1.63	37					−1.64	60	−1.65	10	**2.09**	69		
Neutro-phils markers	*SELL (CD62L)*	−0.25	72	−0.8	57	−0.91	48	−0.63	22	−1.18	7	−0.56	89	**2.84**	77	−0.6	82									−1.19	14		
	*ITGAM*													**1.47**	71														
Cycling cells markers	*ENSCAFG00000030087 (PCALF)*																			**2.75**	96								
	*TOP2A*																			**1.43**	82								
	*ENSCAFG00000013255 (Ki−67)*																			**0.75**	45								
B cells markers	*ENSCAFG00000030258 (BCR)*					−1.51	64	−1.19	47	−2.02	15	−1.99	75			−1.91	81					**5.37**	87	−2.29	39				
	*FCRLA*																					**1.57**	72						
	*CD19*																					**0.77**	59						
Baso-phils markers	*MS4A2*	−0.6	33	−0.51	39	−0.45	56	−0.39	75	−0.49	2			**0.78**	44							−0.33	11	**3.71**	78				
	*KIT*									−0.30	1					**0.42**	81							**3.13**	100				
	*FCER1G*	**0.58**	98	−1.89	21	−2.17	49			−2.19	4			**1.46**	83			**0.5**	94			−2.08	58	**2.21**	92	−2.09	26		

*The avg logFC was calculated by comparing each cluster to all other clusters, overexpressed transcripts are displayed in bold. Only significant data were reported in the table (P < 0.05). DEGs, differentially expressed genes; Avg logFC, average log2 fold; pct, percentage of cells in the cluster expressing the gene; DC, dendritic cells*.

DEGs and biological processes were further compared between clusters sharing the same cell identity, namely macrophages/monocytes, T lymphocytes, epithelial cells and DC, to better characterize each cluster.

The graph-based clustering of merged single-cells identified four transcriptionally distinct clusters of macrophages/monocytes. In this study, *MARCO*, a class A scavenger receptor involved in host defense and demonstrated to be highly expressed in embryonic-derived or alveolar macrophages (AMs) and not expressed in monocyte-derived macrophages was used to identify AMs (25, 27). *MARCO* was overexpressed in the clusters 0, 3, and 5 compared to all remaining clusters (Figure 2 and Supplementary Table 3).

**Figure 3:**
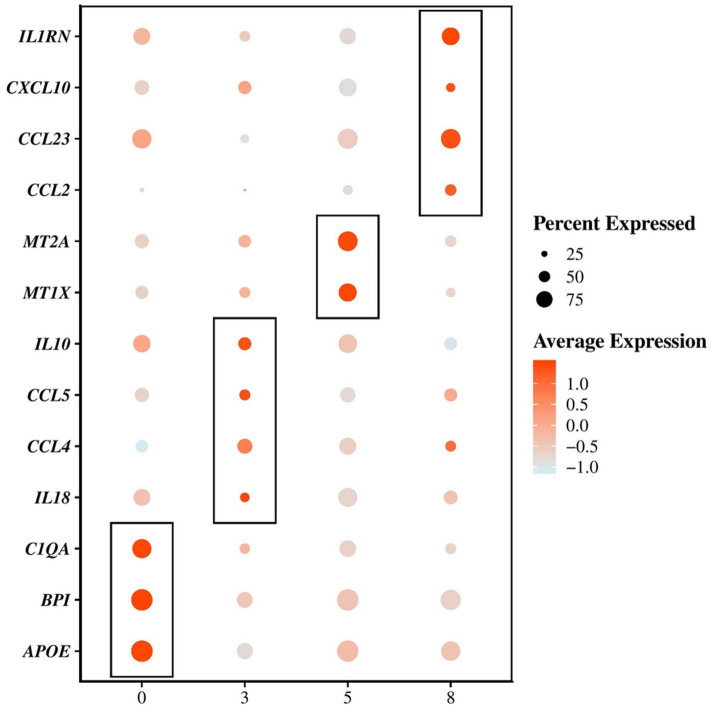
Single-cell mRNA-sequencing based identification of 4 distinct subpopulations of macrophages/monocytes in the bronchoalveolar lavage fluid of dogs. Dot plots showing the average expression of the indicated genes as well as the percentage of cells expressing the genes within each cluster of macrophages/monocytes. An example of transcripts significantly (*P*-value adjusted < 0.05) differentially upregulated (average log2 fold change > 0.5) between the clusters 0, 3, 5, and 8 are depicted.

The first cluster of AMs (cluster 0) represented the majority of the macrophages/monocytes cells and showed a unique transcriptional signature including upregulation of transcripts coding for cell surface markers such as MHC-II molecules (e.g., *DLA-DQA1, DLA-DRA, DLA-DMA*), *CD63*, the Fc fragment of IgG receptor IIIa (*FCGR3A*, encoding *CD16*), the selectin L (*SELL*, encoding *CD62L*), the *CD36* molecule, the *CD68* molecule and the lysosomal associated membrane protein 2 (*LAMP2*) (Supplementary Table 4). Other most upregulated transcripts (average log2 fold change (avg_logFC) > 0.5, *P* < 0.05) included the apolipoprotein E (*APOE*) known as an anti-inflammatory, anti-proliferative and immune-modulatory protein (41) and transcripts involved in the immune response such as for example the bactericidal permeability increasing protein (*BPI*) and the complement C1q A chain (*C1QA*) (Figure 3 and Supplementary Table 4). The principal biological functions exerted by cells in cluster 0 are reported in Table 4.

**Table 4:** Top 10 gene set overlap between significantly upregulated genes in cluster 0, 3, 5 and 8 compared to each other and the gene ontology (GO) biological process gene set.

	**Gene set name**	**Genes in gene set (K)**	**Positive DEGs included**	**Genes in overlap (k)**	**k/K**	**FDR q-value**
Cluster 0 vs. 3, 5 and 8	Myeloid leukocyte activation	650	88	28	0.0431	1.23E-24
	Leukocyte mediated immunity	867	88	30	0.0346	3.06E-24
	Cell activation involved in immune response	705	88	28	0.0397	3.36E-24
	Myeloid leukocyte mediated immunity	550	88	26	0.0473	3.36E-24
	Exocytosis	899	88	29	0.0323	8.10E-23
	Immune effector process	1,253	88	32	0.0255	1.42E-22
	Cell activation	1,424	88	32	0.0225	6.03E-21
	Secretion	1,638	88	31	0.0189	4.86E-18
	Defense response	1,709	88	26	0.0152	2.40E-12
	Innate immune response	984	88	21	0.0213	2.50E-12
Cluster 3 vs. 0, 5 and 8	Response to cytokine	1,192	251	45	0.0378	4.29E-18
	Regulation of immune system process	1,631	251	47	0.0288	1.22E-14
	Positive regulation of protein metabolic process	1,633	251	46	0.0282	3.81E-14
	Cell activation	1,424	251	43	0.0302	3.81E-14
	Cell motility	1,719	251	46	0.0268	1.95E-13
	Response to oxygen containing compound	1,616	251	44	0.0272	4.45E-13
	Locomotion	1,943	251	48	0.0247	6.01E-13
	Defense response	1,709	251	44	0.0257	2.46E-12
	Regulation of cell activation	608	251	27	0.0444	3.06E-12
	Interspecies interaction between organisms	927	251	32	0.0345	7.69E-12
Cluster 5 vs. 0, 3 and 8	Myeloid leukocyte activation	650	62	16	0.0246	1.70E-11
	Myeloid leukocyte mediated immunity	550	62	15	0.0273	1.70E-11
	Exocytosis	899	62	17	0.0189	5.14E-11
	Cell activation involved in immune response	705	62	15	0.0213	2.91E-10
	Leukocyte mediated immunity	867	62	16	0.0185	2.91E-10
	Secretion	1638	62	20	0.0122	2.91E-10
	Cell activation	1,424	62	18	0.0126	2.95E-09
	Immune effector process	1253	62	17	0.0136	3.90E-09
	Cellular homeostasis	971	62	14	0.0144	1.69E-07
	Homeostatic process	1,913	62	18	0.0094	2.57E-07
Cluster 8 vs. 0, 3 and 5	Defense response	1,709	59	20	0.0117	1.22E-09
	Cell motility	1,719	59	19	0.0111	7.91E-09
	Cytokine mediated signaling pathway	787	59	14	0.0178	1.49E-08
	Locomotion	1,943	59	19	0.0098	3.31E-08
	Inflammatory response	722	59	13	0.018	4.87E-08
	Leukocyte migration	488	59	11	0.0225	1.29E-07
	Response to cytokine	1,192	59	15	0.0126	1.29E-07
	Response to bacterium	681	59	12	0.0176	2.45E-07
	Response to biotic stimulus	1,023	59	13	0.0127	1.88E-06
	Regulation of immune system process	1,631	59	15	0.0092	6.45E-06

*Gene set enrichment analysis carried on by computing overlaps between significantly upregulated genes (P < 0.05, avg_logFC > 0.25) and the gene ontology biological process gene set. “Genes in Gene Set” refers to the number of genes in the gene set, “Positive DEGs included” corresponds to the number of positive differentially expressed genes in the cluster of interest compared to the others and “Genes in Overlap” to the number of genes upregulated in the cluster and involved in the biological process. Avg_logFC, average log2 fold change*.

Compared with macrophages composing clusters 0, 5 and 8, the AMs composing cluster 3 overexpressed (avg_logFC > 0.5, *P* < 0.05) transcripts encoding cell surface markers such as the macrophage mannose receptor (*MRC1*, encoding *CD206*), the integrin subunit alpha 5 (*ITGA5*), the scavenger receptor *CD163*, the *CD80* molecule and the *CD83* molecule (Supplementary Table 4). The cells in cluster 3 also largely overexpressed transcripts (avg_logFC > 0.5, *P* < 0.05) encoding cytokines, including the interleukin 18 (*IL18*), the C-C motif chemokine ligand 4 and 5 (*CCL4* and *CCL5*) and the interleukin 10 (*IL10*) (Figure 3 and Supplementary Table 4). Such combination of pro-inflammatory and immunoregulatory cytokines is consistent with the enriched functional properties of the cells in cluster 3 which include regulation of the immune response and cell activation (Table 4).

AMs in cluster 5, in comparison with cells from clusters 0, 3 and 8 also overexpressed transcripts encoding cell surface markers (avg_logFC > 0.5, *P* < 0.05), including the *CD9* molecule, the CD5 molecule like (*CD5L*), *CD68*, the carcinoembryonic antigen related cell adhesion molecule 5 (*CEACAM5*) and the *CD300C* molecule (Supplementary Table 4). The principal functions of AMs composing cluster 5 were quite similar to those associated with AMs in cluster 0. However, those cells seemed to be more involved in cellular homeostasis (Table 4), mostly metal ion homeostasis. Indeed, the most enriched transcripts in the cluster 5 were the metallothionein 1X (*MT1X*) and the metallothionein 2A (*MT2A*) which encode anti-oxidant proteins that are important in the homeostasis of metal in the cell, and in the detoxification of heavy metals (Figure 3 and Supplementary Table 4) (42, 43).

The cells in cluster 8 compared with other clusters did not overexpress the transcript encoding *MARCO* and were not considered as AMs. Overexpressed transcripts coding for surface markers in cluster 8 compared with clusters 0, 3, and 5 included *MHC-II* (*DLA-DQA1*) and *MHC-I* (*DLA-88*) molecules, the tumor necrosis factor superfamily member 13b (*TNFSF13B*), the colony stimulating factor 2 receptor subunit beta (*CSF2RB*), the integrin subunit alpha X (*ITGAX*) and the *CD1e* molecule (Supplementary Table 4). The cells in cluster 8 were characterized by the overexpression (avg_logFC > 0.5, *P* < 0.05) of transcripts encoding cytokines including the interleukin 1 receptor antagonist (*IL1RN*), the C-C motif chemokine ligand 23 (*CCL23*), the C-C motif chemokine ligand 2 (*CCL2*) and the C-X-C motif chemokine ligand 10 (*CXCL10*) (Figure 3 and Supplementary Table 4). The high level of cytokine transcripts in cluster 8 is consistent with the enrichment for processes related to the inflammatory response, the defense response and the response to cytokines (Table 4).

By looking at the DEGs and the GSEA between cluster 1 and cluster 2 corresponding each to T cells (Supplementary Table 5 and Table 5), we were able to characterize cells in cluster 1 as cytotoxic or CD8^+^ T cells. Indeed, the transcripts encoding granzyme B, K, and A (*GZMB, GZMK*, and *GZMA*, respectively) were overexpressed with an avg_logFC > 1 in cluster 1 compared to cluster 2 (Supplementary Table 5). Those genes are expressed by cytotoxic T lymphocytes and natural killer cells (44, 45). Other transcripts with an avg_logFC > 1 in cluster 1 compared to cluster 2 included the killer cell lectin like receptor D1 and K1 (*KLRD1* and *KLRK1*, respectively) also expressed primarily in natural killer cells and CD8^+^ T cells (45, 46). Finally, the *CD8b* molecule was also overexpressed in cluster 1 (Supplementary Table 5).

**Table 5:** Top 10 gene set overlap between significantly upregulated genes in cluster 1 and 2 compared to each other and the gene ontology (GO) biological process gene set.

	**Gene set name**	**Genes in gene set (K)**	**Positive DEGs included**	**Genes in overlap (k)**	**k/K**	**FDR q-value**
Cluster 1 vs. 2	Regulation of immune system process	1,631	24	13	0.008	1.85E-08
	Innate immune response	984	24	11	0.0112	2.20E-08
	Regulation of immune response	1,094	24	11	0.0101	4.58E-08
	Natural killer cell mediated immunity	65	24	5	0.0769	9.46E-07
	Defense response	1,709	24	11	0.0064	3.10E-06
	Regulation of natural killer cell chemotaxis	9	24	3	0.3333	2.26E-05
	Natural killer cell chemotaxis	11	24	3	0.2727	3.81E-05
	Lymphocyte mediated immunity	344	24	6	0.0174	5.41E-05
	Lymphocyte chemotaxis	62	24	4	0.0645	5.41E-05
	Cell activation	1,424	24	9	0.0063	8.11E-05
Cluster 2 vs. 1	Regulation of lymphocyte activation	478	37	12	0.0251	1.38E-10
	Regulation of cell activation	608	37	12	0.0197	8.27E-10
	Regulation of T cell activation	313	37	10	0.0319	8.27E-10
	T cell activation	459	37	11	0.024	8.27E-10
	Regulation of cell death	1,723	37	16	0.0093	2.23E-09
	Lymphocyte activation	721	37	12	0.0166	2.88E-09
	Apoptotic process	1,980	37	16	0.0081	1.30E-08
	Biological adhesion	1,417	37	14	0.0099	2.16E-08
	Cell activation	1,424	37	14	0.0098	2.16E-08
	Leukocyte cell-cell adhesion	336	37	9	0.0268	2.16E-08

*Gene set enrichment analysis carried on by computing overlaps between significantly upregulated genes (P < 0.05, avg_logFC > 0.25) and the gene ontology biological process gene set. “Genes in Gene Set” refers to the number of genes in the gene set, “Positive DEGs included” corresponds to the number of positive differentially expressed genes in the cluster of interest compared to the other and “Genes in Overlap” to the number of genes upregulated in the cluster and involved in the biological process. Avg_logFC, average log2 fold change*.

When comparing cluster 2 to cluster 1, enriched biological processes were more in favor of CD4^+^ T cells, as reported in Table 5. Although classical surface marker of this cell type was not expressed by the cells in our dataset (e.g., *CD4*), the cells in cluster 2 overexpressed transcripts encoding for the interleukin 7 receptor (*IL7R)* and the *CD40* ligand (*Cd40L*) commonly found in CD4^+^ T cells (47, 48). Principal overexpressed transcripts in cluster 2 included *ICOS* (inducible T cell costimulatory) an important costimulatory factor expressed in activated T cells (49–51), *PLAC8* (placenta associated 8), *GATA3* (GATA binding protein 3) and *ANXA1* (annexin A1) (Supplementary Table 5). Those transcripts are associated with the activation of CD4^+^ T cells, T cell differentiation in CD4^+^ T cells and immune response modulation (52–55), which is coherent with the functions of CD4^+^ T cells as reported in Table 5.

Diverse epithelial populations were captured and corresponded to clusters 4 and 12. Cluster 12 was identified as composed by ciliated epithelial cells as it included functions such as cilium movement, microtubule-based process and movement, cytoskeleton and cell projection organization (Table 6). Indeed, the most enriched transcript in cluster 12 compared to cluster 4 included notably the transcripts encoding *SNTN* (sentan, cilia apical structure protein), *DPCD* [deleted in primary ciliary dyskinesia homolog (mouse)], *ROPN1L* (rhophilin associated tail protein 1 like), *SPA17* (sperm autoantigenic protein 17) and *WDR78* (WD repeat domain 78) for example (Supplementary Table 6).

**Table 6:** Top 10 gene set overlap between significantly upregulated genes in cluster 4 and 12 compared to each other and the gene ontology (GO) biological process gene set.

	**Gene set name**	**Genes in gene set (K)**	**Positive DEGs included**	**Genes in overlap (k)**	**k/K**	**FDR q-value**
Cluster 12 vs. 4	Microtubule based process	734	93	14	0.0191	7.90E-06
	Epithelial cilium movement	23	93	5	0.2174	7.90E-06
	Cilium movement	65	93	6	0.0923	2.00E-05
	Cytoskeleton organization	1298	93	17	0.0131	2.00E-05
	Microtubule based movement	277	93	9	0.0325	3.13E-05
	Regulation response to stress	1,497	93	17	0.0114	8.91E-05
	Cell projection organization	1,512	93	16	0.0106	5.00E-04
	Reproduction	1,459	93	15	0.0103	1.49E-03
	Actin filament bundle organization	155	93	6	0.0387	1.59E-03
	Central nervous system development	980	93	12	0.0122	2.54E-03

*Gene set enrichment analysis carried on by computing overlaps between significantly upregulated genes (P < 0.05, avg_logFC > 0.25) and the gene ontology biological process gene set. No overlap was found between the gene set and upregulated genes of the cluster 4 compared to the cluster 12. “Genes in Gene Set” refers to the number of genes in the gene set, “Positive DEGs included” corresponds to the number of positive differentially expressed genes in the cluster of interest compared to the other and “Genes in Overlap” to the number of genes upregulated in the cluster and involved in the biological process. Avg_logFC, average log2 fold change*.

Cells in cluster 7 and 13 were identified as DC. Compared with cells in cluster 13, cells in cluster 7 overexpressed (avg_logFC > 1) transcripts coding for MHC-II and MHC-I molecules (i.e., *HLA-DRB1, HLA-DQA1, DLA-DMA, DLA-DQA1, DLA-DRA, DLA-DOA, DLA-88*, and *DLA-79*) (Supplementary Table 7) and their major functions concerned the activation of immune cells and the defense response.

Overexpressed surface marker transcripts identified in cells of cluster 13 compared to cluster 7 (avg_logFC > 1) included the C-C motif chemokine receptor 7 and the C-X-C motif chemokine receptor 4 (*CCR7* and *CXCR4*, respectively), the *CD83* molecule and the programmed cell death 1 ligand 2 (*PDCD1LG2*) (Supplementary Table 7). The major functions of cells in cluster 13 concerned mostly the regulation of the activation of immune cells (Table 7). Because of their overexpression of *CD83* and *CCR7*, we considered that DC of cluster 13 correspond to mature DC (56, 57).

**Table 7:** Top 10 gene set overlap between significantly upregulated genes in clusters 7 and 13 compared to each other and the gene ontology (GO) biological process gene set.

	**Gene set name**	**Genes in gene set (K)**	**Positive DEGs included**	**Genes in overlap (k)**	**k/K**	**FDR q-value**
Cluster 7 vs. 13	Cell activation	1424	218	59	0.0414	3.15E-31
	Myeloid leukocyte activation	650	218	43	0.0662	4.95E-30
	Myeloid leukocyte mediated immunity	550	218	38	0.0691	5.41E-27
	Cell activation involved in immune response	705	218	41	0.0582	1.26E-26
	Immune effector process	1,253	218	51	0.0407	1.26E-26
	Exocytosis	899	218	44	0.0489	8.25E-26
	Leukocyte mediated immunity	867	218	43	0.0496	1.90E-25
	Secretion	1,638	218	54	0.033	3.62E-24
	Defense response	1,709	218	51	0.0298	1.02E-20
	Regulation of immune system process	1,631	218	46	0.0282	1.96E-17
Cluster 13 vs. 7	Cell activation	1,424	85	23	0.0162	2.66E-10
	Regulation of lymphocyte activation	478	85	14	0.0293	6.34E-09
	Lymphocyte activation	721	85	16	0.0222	6.34E-09
	Regulation of immune system process	1,631	85	22	0.0135	6.34E-09
	Regulation of cell activation	608	85	15	0.0247	6.34E-09
	Regulation of T cell activation	313	85	12	0.0383	6.34E-09
	Response to biotic stimulus	1,023	85	18	0.0176	8.72E-09
	T cell activation	459	85	13	0.0283	2.55E-08
	Cytokine production	759	85	15	0.0198	9.27E-08
	Response to cytokine	1,192	85	17	0.0143	6.25E-07

*Gene set enrichment analysis carried on by computing overlaps between significantly upregulated genes (P < 0.05, avg_logFC > 0.25) and the gene ontology biological process gene set. “Genes in Gene Set” refers to the number of genes in the gene set, “Positive DEGs included” corresponds to the number of positive differentially expressed genes in the cluster of interest compared to the other and “Genes in Overlap” to the number of genes upregulated in the cluster and involved in the biological process. Avg_logFC, average log2 fold change*.

## Discussion

In this paper, we report for the first-time a comprehensive single-cell expression profiling of the canine BALF cells in healthy condition. We were able to cluster cells in 14 distinct subsets identified as macrophages/monocytes, CD8^+^ T cells, CD4^+^ T cells, epithelial cells, ciliated epithelial cells, mature DC and DC, neutrophils, B cells, mast cells and cycling cells.

Until recently, cells of the dog BALF were only characterized by microscopic evaluation or, in rare cases, by flow cytometry. The cell populations identified by these techniques included macrophages, CD4^+^ and CD8^+^ lymphocytes, neutrophils, eosinophils, mast cells and epithelial cells (13–19, 58). With the use of the scRNA-seq, we highlighted the presence of 14 subpopulations of cells using an unbiased technique. We were able to characterized the cells composing those subpopulations in depth and to deduce their main functions based on their transcriptome. In addition to offer a way to overcome the lack of qualitative reagents designed for flow cytometry in dogs, the scRNA-seq allows a better characterization of cell heterogeneity without prior knowledge by highlighting, in better agreement with pulmonary physiology, all cell types and cell functions. Indeed, the scRNA-seq provides comprehensive profiles of cells without limitations due to pre-selected cells by probing a few selected markers (5, 6, 20, 59, 60).

Four subpopulations of macrophages/monocytes were found. Among them, 3 subpopulations corresponded to AMs based on their expression of *MARCO* (25, 27). AMs are the most abundant cells found in the airways in homeostatic conditions. They are self-maintaining with minimal contributions from circulating monocytes in healthy conditions (25, 61, 62). The first subpopulation of AMs, representing the major cell subpopulation, exerted functions involved in immune defense and response. The second was enriched in a combination of pro and anti-inflammatory cytokines transcripts and exerted functions involved in the regulation of the immune response. Finally, the third population had similar functions as the first with more implications in the homeostasis and the detoxification of metal ions. The last subpopulation was not considered as AMs and could correspond to monocytes-derived macrophages or monocytes. Indeed, the cluster was the smallest and expressed macrophages markers but not *MARCO*.

We found a large population of non-ciliated cells and a small population of ciliated cells corresponding to tracheobronchial epithelial cells.

T lymphocytes were subdivided into 2 subpopulations identified as CD8^+^ and CD4^+^ with a majority of CD8^+^ T cells which was already reported in healthy dogs particularly in aged animals (16, 17). Indeed, cells in cluster 1 expressed the *CD8b* molecule. However, cells in cluster 2 did not express neither *CD8* nor *CD4* while they overexpressed markers associated with classical CD4^+^ T cells such as *GATA3, IL7R* and the *CD40* ligand (47, 48, 53, 54). The absence of *CD4* mRNA expression could be due to weakness or absence of transcription of this protein (60). Indeed, in dogs, a population of CD8^−^CD4^−^ T cell has been described representing ~15% of the TCRαβ^+^ T cells in the lung, also expressing *GATA3* (63). Cells from cluster 2 cells possibly belong to this population.

B cells were identified using *BCR, FCRLA* and *CD19* markers (37–39). *CD19* has only recently been described as B cell marker in dogs (39) which highlights the benefice of the scRNA-seq for the identification of new surface markers to better isolate different cell types (5, 6). Common B cell surface markers used and described in dogs include *CD21* and *CD79A* (64). In this study, *CD21* mRNA was not detected which could be due to absent or weak transcription of this protein (60). *CD79A* was expressed in B cells but its expression was low and it was not significantly differentially expressed in the cluster 10 compared to others.

The identified granulocytic populations included neutrophils and mast cells. Basophils and mast cells shared common markers including *MS4A2, KIT* and *FCER1G* used in our study (40). However, overexpressed DEGs in cluster 11 cells also included chymase (encoding *CMA1*) and tryptase enzymes (encoding *PPSAB1* and *TSP2*) which are almost entirely mast cell-specific (40, 65, 66). The cells in cluster 11 also expressed *CD63* which is considered as one of the most useful markers of mast cell and basophil activation (40, 66). We did not identify mast cells in BALF 1 which is probably due to the small proportion of that cell type into BALF samples (13). Indeed, it represents only 1.0% of the total cells recovered in this analysis and it is possible that rare cell populations may not be properly captured with the 10X Genomics Chromium system (60). We were not able to identify a cluster of eosinophils, which are normally present in dog BALF specimens (13). Although the number of eosinophils found in the BALF of healthy dogs is rather low (13) and may not be properly captured, their total absence from our dataset is most likely related to their high content of RNase (67) inducing the rapid degradation of mRNA, thus preventing their detection by scRNA-seq.

Finally, two subpopulations of DC were also found one being identified as mature DC because of its higher expression of *CD83* and *CCR7* (56, 57).

The use of the scRNA-seq in dogs has some limitations. The principal impediment to apply scRNA-seq to canine samples is the necessity to map sequenced RNAs on a sufficiently well-annotated database to be able to identify genes. The percentage of reads mapped confidently to the transcriptome in this study was considered as low (~28%) as it is expected to be > 30%^1^. This can be due to a poor annotation of the reference transcriptome (overlapping genes for example), but could also be related to a poor library, sequencing or reads quality ^1^. However, despite this suboptimal mapping, we were able to identify cell clusters and deduce clusters principal functions based on the cell transcriptome obtained. Another limitation is the lack of information for the identification of specific cell markers in dogs. For example, in the 4 clusters identified as macrophages/monocytes, AMs were recognized only by their expression of *MARCO*. In the literature in human and mouse, the expression of *SIGLECF, MERTK, CD14, CCR2*, and *Ly-6c* are commonly used to distinguish AMs from monocytes and monocyte-derived macrophages (8, 21, 26, 27, 68, 69). However, those markers were not detected in our dataset. The use of the 10X Genomics Chromium system although being unbiased, time saving and allowing high throughput and high-resolution transcriptomic analysis, also implies that rare cell populations may not be properly captured and that sensitivity is reduced decreasing the detection of weakly expressed genes (60). It is possible that common markers used to identify different cell types are only weakly expressed making cell populations difficult to identify. Finally, a limited number of dogs was used in the study. Indeed, as the use of the scRNA-seq is quite expensive, only 4 BALF samples were analyzed with a relatively low median number of cells and reads per sample (~1,300 cells and ~54,000 reads, respectively). The 4 selected dogs included young to old adult dogs from 4 breeds differing in size and body conformation, in order to be, as much as possible, representative of the whole healthy canine population, even if no males were sampled. However, we don't expect the sex to alter BALF cells transcriptome. While it has been shown that the age could alter the cell proportions in the BALF from healthy dogs (70), we are not aware of studies assessing its effect on BALF cells transcriptome either. To our knowledge, no study has specifically investigated the effect of the sex and the breed on canine BALF cells proportions and transcriptome. Besides, in the present study, cells coming from each of the four BALF specimens were present in nearly all identified clusters indicating that similar cell populations were present in all dogs.

## Conclusion

ScRNA-seq is a new technique which enables unbiased, high throughput and high-resolution transcriptomic analysis and which can be used to identify cell populations in the BALF of healthy dogs. In this study, we provide a comprehensive single-cell transcriptome tool. It represents a highly informative dataset for the identification and subsequent interpretation of cell populations and molecular signatures alterations in lung diseases in dogs.

## Data Availability Statement

The datasets presented in this study can be found in online repositories. The names of the repository/repositories and accession number(s) can be found below: https://www.ebi.ac.uk/arrayexpress/, E-MTAB-9265.

## Ethics Statement

The animal study was reviewed and approved by Ethical committee of the University of Liège, Liège, Belgium. Written informed consent was obtained from the owners for the participation of their animals in this study.

## Author Contributions

AF, CC, and FB designed the study. AF, DP, LF, and CC conducted experiments and acquired the data. AF and DP analyzed the data. FB, CD, and TM provided their expertise in lung immune cells. AF wrote the manuscript. All authors interpreted the results and reviewed and approved the final version of the manuscript.

## Conflict of Interest

The authors declare that the research was conducted in the absence of any commercial or financial relationships that could be construed as a potential conflict of interest.

## Footnotes

^1^https://support.10xgenomics.com/single-cell-gene-expression/software/pipelines/latest/troubleshooting#alerts (accessed March 16, 2020).

## Abbreviations

ScRNA-seq: Single-cell RNA sequencing
BALF: Bronchoalveolar lavage fluid
TCC: Total cell count
DCC: Differential cell count
UMI: Unique Molecular Identifier
PCA: Principal component analysis
t-SNE: t-distributed stochastic neighbor embedding
DEGs: Differentially expressed genes
GSEA: Gene set enrichment analysis
GO: Gene ontology
DC: Dendritic cells
AMs: Alveolar macrophages
Avg_logFC: Average log2 fold change.
