# Supplementary material for: Characterization of the Bronchoalveolar Lavage Fluid by Single Cell Gene Expression Analysis in Healthy Dogs: A Promising Technique
Source: Front Immunol. 2020 Jul 30;11:1707. doi: 10.3389/fimmu.2020.01707 (PMC7406785; doi:10.3389/fimmu.2020.01707)
Supplement: Supplementary file 8 [file Data_Sheet_1.docx]

Supplementary Material

## Supplementary Figures


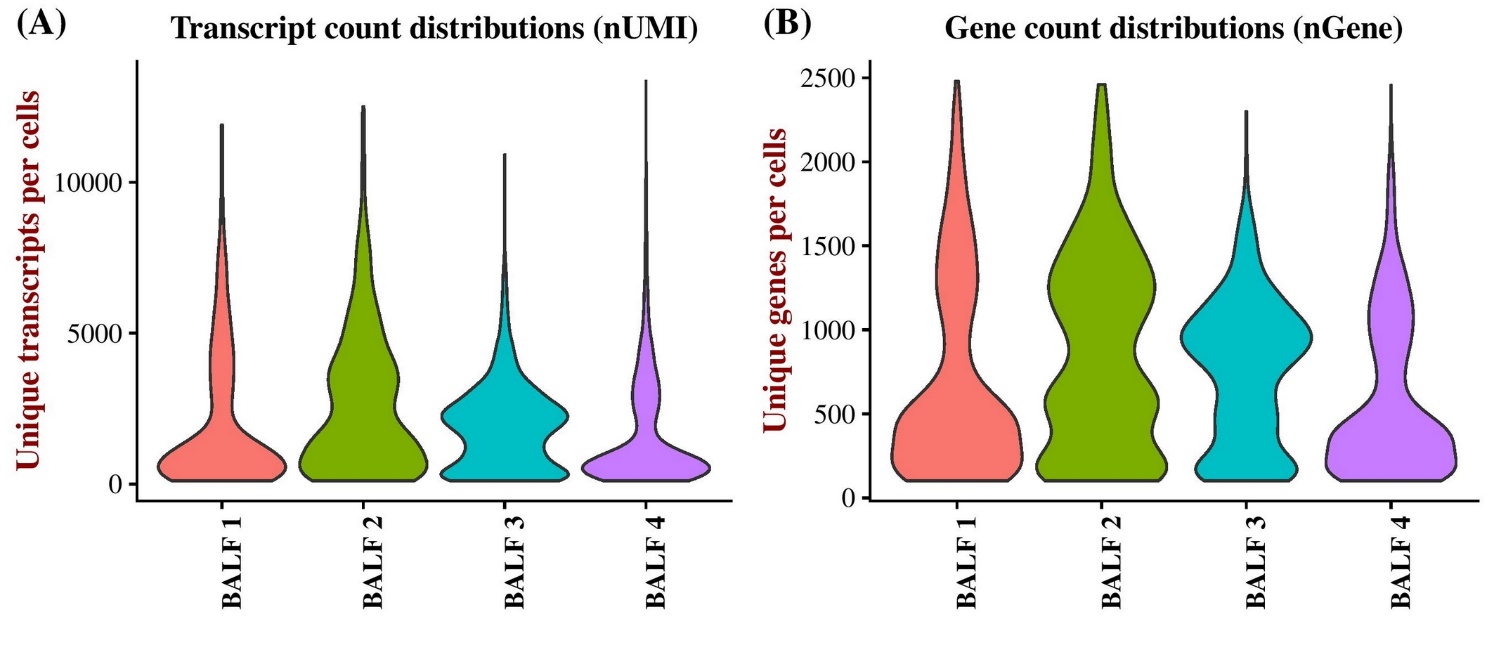


**Supplementary Figure 1.** **Dataset quality control.** (A) Unique transcript (nUMI) distributions; (B) Unique gene (nGene) distributions. Colors match across BALF specimens. BALF 1, female Yorkshire terrier of 11-year-old; BALF 2, female French bulldog of 4-year-old; BALF 3, female West Highland white terrier of 9-year-old; BALF 4, female Australian shepherd of 6-year-old.
